# Anti-Fibrotic Efficacy of Apigenin in a Mice Model of Carbon Tetrachloride-Induced Hepatic Fibrosis by Modulation of Oxidative Stress, Inflammation, and Fibrogenesis: A Preclinical Study

**DOI:** 10.3390/biomedicines11051342

**Published:** 2023-05-02

**Authors:** Maryam Melaibari, Huda M. Alkreathy, Ahmed Esmat, Nisreen A. Rajeh, Rasheed A. Shaik, Anwar A. Alghamdi, Aftab Ahmad

**Affiliations:** 1 Department of Pharmacology, Faculty of Medicine, King Abdulaziz University, Jeddah 21589, Saudi Arabia; maryam.melaibari@gmail.com (M.M.); aameer@kau.edu.sa (A.E.); 2 Department of Pharmacology and Toxicology, Faculty of Pharmacy, Ain Shams University, Cairo 11566, Egypt; 3 Department of Clinical Anatomy, Faculty of Medicine, King Abdulaziz University, Jeddah 21589, Saudi Arabia; nrajeh@kau.edu.sa; 4 Department of Pharmacology and Toxicology, Faculty of Pharmacy, King Abdulaziz University, Jeddah 21589, Saudi Arabia; rashaikh1@kau.edu.sa; 5 Health Information Technology Department, The Applied College, King Abdulaziz University, Jeddah 21589, Saudi Arabia; nloalgamdi7@kau.edu.sa; 6 Pharmacovigilance and Medication Safety Unit, Center of Research Excellence for Drug Research and Pharmaceutical Industries, King Abdulaziz University, Jeddah 21589, Saudi Arabia

**Keywords:** liver, fibrosis, Apigenin, oxidative stress, inflammation, angiogenesis, VEGF

## Abstract

Background: Hepatic fibrosis is a major health problem all over the world, and there is no effective treatment to cure it. Hence, the current study sought to assess the anti-fibrotic efficacy of apigenin against CCl_4_-induced hepatic fibrosis in mice. Methods: Forty-eight mice were put into six groups. G1: Normal Control, G2: CCl_4_ Control, G3: Silymarin (100 mg/kg), G4 and G5: Apigenin (2 &20 mg/Kg), G6: Apigenin alone (20 mg/Kg). Groups 2, 3, 4, and 5 were given CCl_4_ (0.5 mL/kg. i.p.) twice/week for six weeks. The level of AST, ALT, TC, TG, and TB in serum and IL-1β, IL-6, and TNF-α in tissue homogenates were assessed. Histological studies by H&E staining and Immunostaining of liver tissues were also performed. Results: The CCl_4_-challenged group showed increased serum AST (4-fold), ALT (6-fold), and TB (5-fold). Both silymarin and apigenin treatments significantly improved these hepatic biomarkers. The CCl_4_-challenged group showed reduced levels of CAT (89%), GSH (53%), and increased MDA (3-fold). Both silymarin and apigenin treatments significantly altered these oxidative markers in tissue homogenates. The CCl_4_-treated group showed a two-fold increase in IL-1β, IL-6, and TNF-α levels. Silymarin and apigenin treatment considerably decreased the IL-1β, IL-6, and TNF-α levels. Apigenin treatment inhibited angiogenic activity, as evidenced by a decrease in VEGF (vascular endothelial growth factor) expression in liver tissues, and a decline in vascular endothelial cell antigen expression (CD34). Conclusions: Finally, these data collectively imply that apigenin may have antifibrotic properties, which may be explained by its anti-inflammatory, antioxidant, and antiangiogenic activities.

## 1. Introduction

Hepatic fibrosis is a healing mechanism of the liver that manifests after a sustained liver injury. The extracellular matrix (ECM) accumulates in place of hepatocytes during this phase of healing [1,2]. Over two million people die from liver disease each year in the world, including one million who die from cirrhosis complications, one million from viral hepatitis, and one million from hepatocellular carcinoma. Together, cirrhosis and liver cancer cause 3.5% of all deaths globally, ranking as the 11th and 16th most frequent causes of death worldwide, respectively [3]. Liver diseases are classified as fatty liver (hepatic steatosis), fibrosis, cirrhosis, or cancer based on the pattern of hepatocellular injury [4]. One of the most prevalent chronic liver diseases is fatty liver, commonly referred to as hepatic steatosis, which affects around one-fourth of the world’s population. By 2030, it is expected to become the main reason people need liver transplants, overtaking other liver diseases. This will have a big effect on the health of the whole world. Approximately 40% of patients with hepatic steatosis disease will develop liver fibrosis [5]. In addition, if advanced fibrosis is not treated, it can lead to irreversible cirrhosis, which can cause hepatic failure or cancer [6]. Liver cirrhosis is characterized by a high risk of mortality, and transplantation is the only viable treatment [7]. Hepatic fibrosis is a pathological process of healing from chronic liver damage and is characterized by abnormal connective tissue growth in the liver induced by many pathogenic components. Hepatic fibrosis was once thought to be irreversible due to the accumulation of collagen and the collapse of the parenchyma. In reality, hepatic fibrosis is reversible, and liver damage frequently includes an HF process as the liver heals and repairs itself. Li et al. (2022) reported that hepatic fibrosis will lead to permanent cirrhosis or even liver cancer if the causes of damage are not eliminated over a long period of time [8]. Recent research has indicated that the early phases of liver diseases, steatosis and fibrosis, can be cured [9,10]. Both oxidative stress and inflammation are linked to fibrotic diseases [11]. A healthy liver can eliminate various oxidants through both enzyme- and non-enzyme-based reactions. Oxidative stress has a significant impact on liver fibrosis and liver damage. A discrepancy among the pro-oxidant and antioxidant cellular components is directly associated with the generation of reactive nitrogen species and reactive oxygen species. However, oxidative stress reduces the antioxidant capacity of the liver [12]. Consequently, reactive free radicals destroy essential biological molecules and activate an inflammatory response, which ultimately causes cell necrosis [13].

Hepatocytes, hepatic stellate cells (HSC), Kupffer cells, and hepatic sinusoidal endothelial cells are some of the cells that contribute to the initiation and development of fibrosis. Hepatic stellate cells are primarily responsible for fibrogenesis. Apoptotic bodies and other tissue factors released by damaged hepatocytes activate macrophages and chemokines. Furthermore, Kupffer cells, liver-specific macrophages, will produce pro-inflammatory cytokines. Non-alcoholic fatty liver disease is primarily caused by the release of pro-inflammatory cytokines from Kupffer cells, like TNF-α, IL-1β, and IL-6 [14,15]. These cytokines have a relationship with the activation and growth of HSCs, the main fibrogenic cells in hepatic fibrosis [16,17]. Moreover, ECM proteins can trigger cytokines and chemokines, amplifying inflammatory activity [18]. Pathological angiogenesis is a crucial component of chronic wound healing in the body [19]. Many investigations have confirmed the link between angiogenesis, hypoxia, and liver fibrosis [20,21]. Due to the high disposition of ECM in the fibrotic liver, venous resistance exceeds compensatory capability and restricts oxygen supply (hypoxia). Recent studies have shown that hypoxia greatly accelerates the progression of liver fibrosis. Hypoxia activates the transcription of proangiogenic genes like vascular endothelial growth factors [22,23]. Consequently, VEGF binds to receptors on endothelial cells (EC) to regulate the formation and proliferation of vascular tissues [24]. Nevertheless, persistent hypoxia enhances collagen synthesis and fibrosis by overproducing proangiogenic factors [21]. Antifibrotic treatments with mild side effects are not yet available for clinical use. The natural products are safer, more effective hepatoprotective drugs [25,26].

Apigenin is a flavone with anti-inflammatory and antioxidant properties [27,28,29]. Notably, apigenin suppresses angiogenesis in diverse disease models by inhibiting VEGF expression [30,31]. It has an excellent safety profile and no significant toxicities even at high doses [32]. Remarkably, apigenin is reported to experimentally reduce induced liver fibrosis by inhibiting angiogenesis [33].

Overall, fibrosis and subsequent organ failure account for at least one-third of all disease-related mortality worldwide. Hence, deciphering the molecular mechanism of liver fibrosis and determining crucial treatment targets are critical issues that must be addressed promptly. The development and progression of liver fibrosis are greatly influenced by oxidative stress, inflammation, and angiogenesis. This study evaluated the anti-fibrotic potential of the dietary flavonoid “apigenin” to address this problem. A mouse model of carbon tetrachloride (CCl_4_)-induced hepatic fibrosis has been widely used to produce liver fibrosis and is one of the most useful models for studying the underlying molecular causes of liver fibrosis. Therefore, this study was designed to explore the anti-fibrotic efficacy using the CCl_4_ model and the specific molecular mechanism of apigenin with respect to the modulation of oxidative stress, inflammation, and fibrogenesis. To better understand the angiogenesis pathway, immunohistochemical detection of vascular endothelial growth factor (VEGF) and vascular endothelial cell antigen (CD34) has been assessed.

## 2. Materials and Methods

### 2.1. The Chemicals and Drugs

Merck^®^ (St Louis, MO, USA) supplied all drugs and chemicals, including apigenin (Cat. #: 178278), silymarin (Cat. #: S0292), CCl_4_ (Cat. #: 270652), and propylene glycol (PG) (Cat. #: PHR1051). The rest of the chemicals were of the purest and best quality obtained from commercial companies.

### 2.2. Animals

Swiss albino (SWR) male mice weighing 30 ± 5 g were obtained from the animal breeding house at the KFMRC (King Fahad Medical Research Centre) of King Abdulaziz University, Jeddah, Saudi Arabia (KSA). Mice were kept at 25 °C in an air-conditioned room with a persistent light/dark cycle. Mice received unlimited clean water and standard feed. The experimental plan was approved by the institutional Ethical Committee for Research at KAU (Approval No. 518-18).

### 2.3. Experimental Protocol

Forty-eight mice were acclimatized and arbitrarily segregated into six groups with eight mice in each. Mice were treated for six weeks as per the following schedule. Group 1 (Normal Control Group): The mice received 2 mL/kg of corn oil by intraperitoneal route two times weekly on alternating days, and 7 mL/kg of propylene glycol (PG) via oral gavage (o.g.) three times weekly. Group 2 (toxic control group): The mice received 0.5 mL/kg of CCl_4_ and a corn oil (1:4) mixture by intraperitoneal route two times per week, and PG (7 mL/kg via oral gavage (o.g.)) three times weekly, alternating with CCl_4_. Group 3 (CCl_4_ + silymarin-treated group): The mice received 0.5 mL/kg of CCl_4_ and a corn oil (1:4) mixture by intraperitoneal route two times per week, and silymarin (100 mg/kg) dissolved in PG via oral gavage (o.g.) three times weekly, alternating with CCl_4_. Group 4 (CCl_4_ + treated with low dose of apigenin): The mice received 0.5 mL/kg of CCl_4_ and a corn oil (1:4) mixture by intraperitoneal route two times per week, and apigenin (2 mg/kg) dissolved in PG via oral gavage (o.g.) three times weekly on alternate days with CCl_4_. Group 5 (CCl_4_ + treated with high dose of apigenin): The mice received 0.5 mL/kg of CCl_4_ and a corn oil (1:4) mixture by intraperitoneal route two times per week, and apigenin (20 mg/kg) dissolved in PG via oral gavage (o.g.) three times weekly on alternate days with CCl_4_. Group 6 (apigenin-alone treated group): Mice were given apigenin (20 mg/kg, o.g.) three times weekly, alternated with corn oil (2 mL/kg, i.p.) two times weekly. Injections of CCl_4_ (0.5–0.7 mL/kg, i.p.) dissolved in a corn oil mixture at a ratio of 1:4, given twice weekly for six weeks, could cause liver fibrosis in mice [34]. Using the body surface area index, the low dose of apigenin (2 mg/kg) was found to be the same as the daily recommended amount of flavonoids for humans. Ullah et al., 2020, reported that flavonoids have anticancer, antioxidant, anti-inflammatory, and immune-modulating effects [35]. In an acute toxicity study on male Swiss mice, apigenin was found to impair liver function and likely be hepatotoxic at doses higher than 50 mg/kg, which is far higher than the doses selected in this investigation [36]. Nevertheless, silymarin (100 mg/kg) was similar to what was described in the published literature on experimentally induced liver fibrosis [37,38]. As reported in previous studies, there was no toxicity or hepatoprotection from the given volume of propylene glycol (10 mL/kg) [39,40]. At the termination of the study, mice were anesthetized by ether inhalation, and blood samples were taken from the retro-orbital plexus, carefully centrifuged, and kept at −30 °C. Afterwards, all mice were then sacrificed by a simple cervical dislocation technique, and their liver samples were carefully harvested. A part of the liver samples from each group was then properly preserved in formalin buffered saline (10%) for immuno-histochemical and histopathological analyses. The remaining liver tissues were minced into small pieces and thoroughly rinsed in ice-cold phosphate buffered solution (PBS) (pH 7.4) to get rid of any excessive blood. The liver tissues were then weighed and homogenized with a suitable amount of PBS in an ice-cold IKA T-25 tissue homogenizer to produce a 1:10 homogenate. The homogenates are then centrifuged for a duration of five minutes at 5000× *g* to obtain the supernatant liquid, which is subsequently kept at −80 °C for use in future biochemical investigations.

### 2.4. Evaluation of Biomarkers of Liver Functions

The serum levels of aspartate aminotransferase (AST) and alanine aminotransferase (ALT), total cholesterol (TC), triglycerides (TG), and total bilirubin (TB) were determined using biochemical kits obtained from Bio-diagnostic^®^ for the assessment of the liver functions (Giza, Egypt).

### 2.5. Histopathological Assessment of the Liver Tissues

The tissue fixation, staining, and histological examination of liver tissues were done according to the standard procedure [41]. Briefly, the samples of liver tissue were preserved in a 10% buffered formalin solution. The paraffin blocks of preserved liver tissues were prepared. The cutting of the paraffin-embedded liver tissues was carried out with a microtome. The four-micron (4 µm) thick sections were prepared, and these liver sections were transferred onto clean glass slides and then properly stained using different types of staining, such as haematoxylin-eosin (H&E) for routine histopathology and Masson’s Trichrome staining to confirm the magnitude of the collagen fibers in the liver tissues. Finally, the stained slides were carefully examined and photographed under ×100 magnification using a light microscope (NikonEclipse 50i, Nikon Corporation, Tokyo, Japan).

### 2.6. Assessment of Oxidative Stress Biomarkers

A liver piece was thoroughly homogenized in a phosphate buffer solution (0.1 M; pH 7.4) and then centrifuged for a duration of fifteen minutes at 10,000 rpm while maintaining a 4 °C temperature. The clear supernatant was separated, and it was used for the estimation of antioxidant enzymes like reduced glutathione (GSH), catalase (CAT), and hepatic concentration of malondialdehyde (MDA) as a marker of lipid peroxidation (LPO) by the method of Amir et al., 2016 [42]. The activities of these antioxidant enzymes were determined in the supernatant with the help of biochemical commercial kits obtained from Bio Diagnostic^®^ (Giza, Egypt). The obtained results were finally expressed per mg of tissue protein.

### 2.7. The Assessment of Inflammatory Biomarkers

The hepatic concentration of inflammatory biomarkers like Interleukin-1β (IL-1β), Interleukin-6 (IL-6), and Tumor Necrosis Factor Alpha (TNF-α) in tissue homogenates was determined by Enzyme linked immunosorbent assay (ELISA) kits (Elabscience^®^, Houston, TX, USA) by following the manufacturer’s instructions. The obtained data were precisely expressed per mg of tissue protein.

### 2.8. Immunohistochemical Assessment of Angiogenic Biomarkers

Immunostaining was carried out according to Buchwalow and Böcker (2010) to detect the constituents of rabbit polyclonal antibodies for both VEGF (Cat. #: ab53465, Abcam plc., Cambridge, UK) and CD34 (Cat. #: ab185732, Abcam plc., Cambridge, UK) [43]. First, four-micron-thick sections were sliced from the paraffin blocks, deparaffinized, and rehydrated using xylene then ethanol solutions. Then, a heat-induced epitope retrieval method was used to retrieve the antigen. Slides were then blocked in a normal serum (10%) containing 1% bovine serum albumin (BSA) in tris-buffered saline for two hours after being rinsed in TBS plus (0.025%) triton X-100 for 10 min with gentle agitation.

In the following step, slides were immunostained with one of the targeted rabbit polyclonal antibodies at 1 µg/mL concentration, which was diluted in TBS with 1% BSA and then incubated for an overnight period at the temperature of 4 °C. The following day, the slides were gently agitated for 10 min while being properly washed with TBS plus (0.025%) Triton X-100. Slides were then exposed to goat anti-rabbit HRP-linked secondary antibody (Cat. #: ab205718), and the slides were yet again incubated at normal room temperature for a period of one hour. After a quick second wash, a substrate solution (0.02%) of diaminobenzidine (DAB) containing (0.01%) hydrogen peroxide (H_2_O_2_) was added to the slides and incubated for 5 min, producing a brown product at the site of the desired antigen. Then, haematoxylin was used as a counterstain, and slides were then dehydrated, cleared, and covered with a glass slip. Finally, positive slides were visualized under an X-100 magnification light microscope (Nikon Eclipse 50i, Nikon Corporation, Tokyo, Japan). Quantitative analysis was carried out as Optical Density (OD), using ImageJ software (1.48a, NIH, Bethesda, MA, USA).

### 2.9. Protein Determination

The total protein content of tissue homogenates was assessed by the bicinchoninic acid (BCA) method using commercial kits (Thermo Scientific™ Pierce™, Cat. #:23225, Rockford, IL, USA).

### 2.10. Statistical Analysis

The one-way analysis of variance (ANOVA) and Tukey’s post-hoc tests were used to conduct the statistical analysis. The obtained data were presented as mean ± S.D., and a *p*-value ≤ 0.05 was then considered as statistical significance. The statistical analyses were accomplished with the help of the GraphPad Instat^®^ software package (Version-3.06). Graphs were created using GraphPad-Prism^®^ software (GraphPad-Software, LLC, San Diego, CA, USA, Version-8).

## 3. Results

### 3.1. Assessment of Liver Functions

There was a substantial increase in the levels of serum AST (4-fold) and ALT (6-fold) in the CCl_4_-challenged group in comparison to the normal control group. In addition, TC and TG were also significantly elevated by almost twofold and TB by more than fivefold in the CCl_4_-challenged group in comparison with the normal control group. In contrast, treatment with silymarin significantly reduced the elevated serum levels of ALT, AST, TC, and TG as compared to the CCl_4_-challenged group. Interestingly, the effect of silymarin on almost all liver function biomarkers was found to be significantly superior to the apigenin-treated groups. Both doses of apigenin (2 mg/kg and 20 mg/kg) exhibited significant dose-dependent effects on the level of serum AST and ALT activities as compared with the CCl_4_-intoxicated group. However, the apigenin (2 mg/kg) did not significantly alter the level of serum TC and TG levels as compared with the CCl_4_-intoxicated group, while it significantly decreased TB concentrations by 38% compared with the CCl_4_-challenged group. Nevertheless, apigenin at 20 mg/kg significantly reduced the serum levels of TC, TG, and TB as compared to the CCl_4_-challenged group. Remarkably, apigenin-alone-treated animals revealed no statistically significant differences from the normal control group in any biomarkers (Table 1).

### 3.2. Findings of Histopathological Analysis

#### 3.2.1. Haematoxylin and Eosin (H&E)

**Figure 1:**
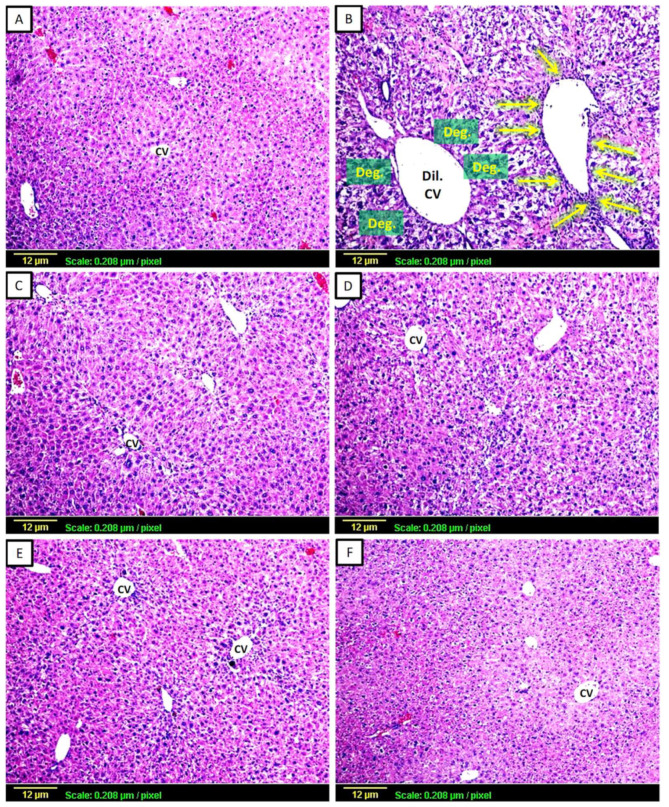
Representative photomicrographs of liver sections stained by H&E (X100). (**A**) Control group showing normal hepatocellular architecture; (**B**) CCl_4_-challenged group showing dilated central vein (Dil. CV) with cell degeneration (Deg.), vacuolization, and inflammatory cell infiltration around the portal vein (yellow arrows), (**C**) CCl_4_ + silymarin-treated group showing minimal changes in hepatic tissue, (**D**) CCl_4_ + Low-dose apigenin-treated group showing moderate centrilobular degeneration, (**E**) CCl_4_ + High-dose apigenin-treated group showing minimal changes in cellular architecture with no infiltration of inflammatory cells, (**F**) Apigenin-alone-treated group showing normal hepatocellular architecture.

To further confirm the induction of hepatotoxicity and the effects of various treatments on hepatotoxicity, as shown in Figure 1, histopathological evaluation of the hepatic tissues of all treated groups was performed. The normal control group’s liver sections showed normal hepatocellular structural architecture, vesicular nuclei, and eosinophilic cytoplasm (Figure 1A). Conversely, sections from the CCl_4_-challenged group displayed a clear dilated central vein with cell degeneration and vacuolization. Moreover, the area around the portal vein also displayed significant inflammatory cell infiltration (Figure 1B). However, the CCl_4_-challenged rats treated with silymarin showed minor alterations to the hepatic tissue and no infiltration of inflammatory cells (Figure 1C), while the group treated with low doses of apigenin (2 mg/kg) showed moderate centrilobular degeneration (Figure 1D). Furthermore, only minor alterations and no inflammatory cell infiltration were visible in the liver tissue of the CCl_4_-challenged rats treated with high doses of apigenin (20 mg/kg) (Figure 1E). The group that was just given apigenin did not exhibit any changes to the typical hepatocellular architecture (Figure 1F).

#### 3.2.2. Masson’s Trichrome

**Figure 2:**
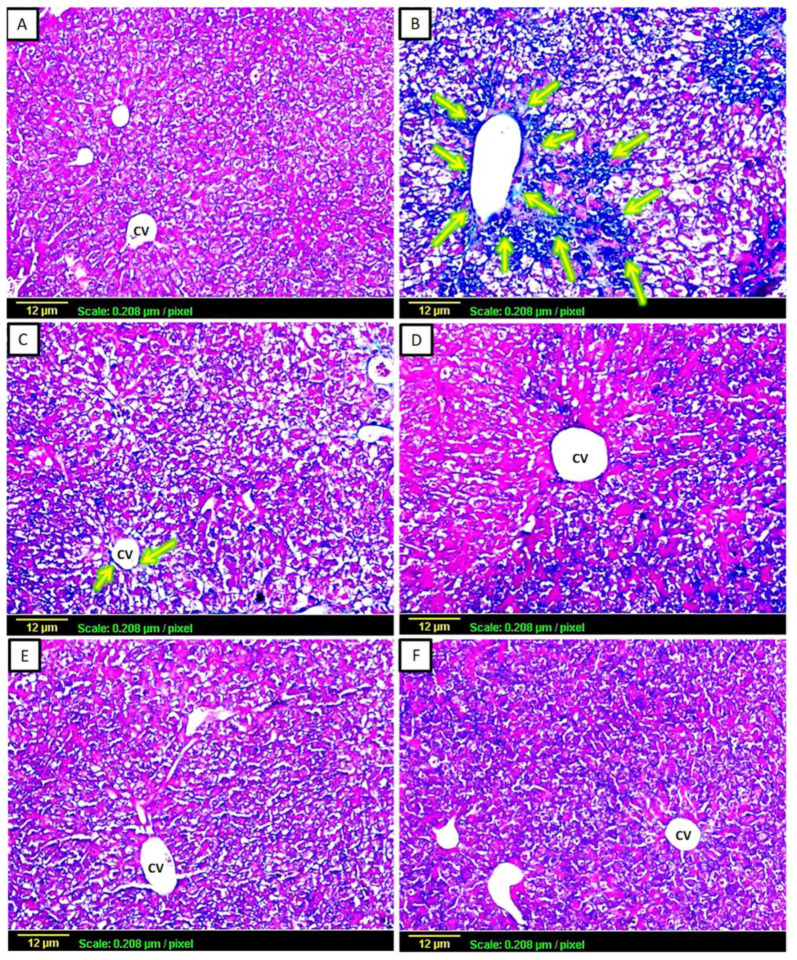
Representative photomicrographs of liver sections stained by Masson’s trichome (×100). Collagen can be visualized by the blue color of stains: (**A**) Control group showing normal degree (+) of collagen fibers; (**B**) CCl_4_-challenged group showing extensive interlobular collagen deposition around the portal vein, appearing as intense (++++) blue-stained content in the tissue (arrows); (**C**) CCl_4_ + silymarin-treated group showing moderate (+++) collagen fibers surrounding the central vein (arrows), (**D**) CCl_4_ + low-dose apigenin-treated group shows a moderate degree (+++) of collagen fibers; (**E**) CCl_4_+high-dose apigenin-treated group shows minimal (++) collagen fiber content; (**F**) apigenin-alone-treated group shows a normal degree (+) of collagen fibers.

This type of staining was used for the histological evaluation of liver fibrosis, as collagen fibers appeared in blue (Figure 2). In the control group sections, there were normal levels of collagen fibers throughout the hepatic tissue (Figure 2A). In contrast, the CCl_4_-challenged group revealed extensive interlobular collagen deposition. In addition, intense blue-stained content was appearing around the central vein (Figure 2B). CCl_4_-intoxicated animals treated with silymarin or low-dose apigenin (2 mg/kg) demonstrated a moderate degree of collagen fibers surrounding the central vein (Figure 2C,D). In contrast, the animals treated with large doses of apigenin (20 mg/kg) showed minimal collagen fiber content in the tissue (Figure 2E), while the apigenin-only treated group exhibited a normal degree of collagen fibers in the hepatic tissue, the same as the control group (Figure 2F).

### 3.3. Evaluation of Oxidative Stress Biomarkers

**Figure 3:**
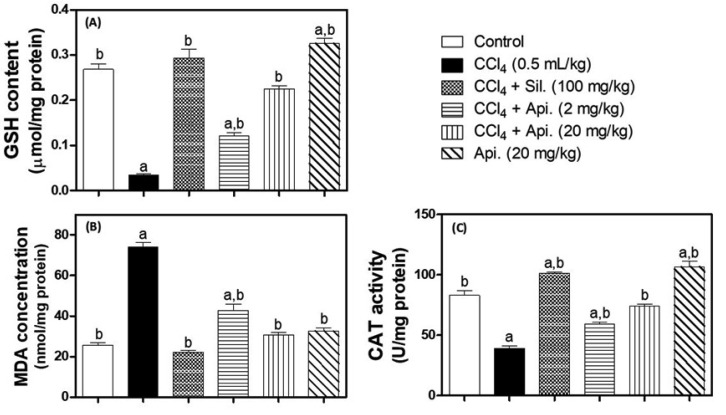
The effect of apigenin on markers of oxidative stress: GSH (Panel (**A**)), MDA (Panel (**B**)), and CAT (Panel (**C**)) in mice with CCl4-induced fibrosis of the liver. The data are presented as Mean ± S.D. (n = 8). **a**: significantly different from the corresponding control at *p* < 0.05; **b**: significantly different from the corresponding CCl_4_-challenged group at *p* < 0.05; **CCl_4_**; carbon tetrachloride, **Sil**.; Silymarin, **Api.**; Apigenin.

MDA concentrations, GSH content, and CAT activity were all analyzed to determine the level of oxidative stress (Figure 3). The administration of CCl_4_ markedly reduced CAT activity and GSH content by 89% and 53%, respectively. Moreover, the administration of CCl_4_ also increased the MDA concentration by approximately threefold in comparison with the normal control group. Conversely, silymarin treatment was able to restore GSH and MDA levels that did not differ significantly from the normal control group. Moreover, CAT activity was 22% higher in the silymarin treatment group of rats in comparison with the animals in the normal control group. Similarly, treatment with apigenin (2 mg/kg and 20 mg/kg) substantially increased GSH, MDA, and CAT depending on the dose in comparison to the CCl_4_-challenged group. Moreover, apigenin treatment (20 mg/kg) was effective in enhancing all measured oxidative biomarkers to the point that they did not statistically differ from the values of the normal control group. Surprisingly, apigenin-alone-treated animals demonstrated statistically significant increases in GSH content by (22%) and CAT activity by 29% compared to the normal control group. However, apigenin alone had no discernible impact on MDA levels in comparison with the normal control group.

### 3.4. Assessment of Inflammatory Biomarkers

**Figure 4:**
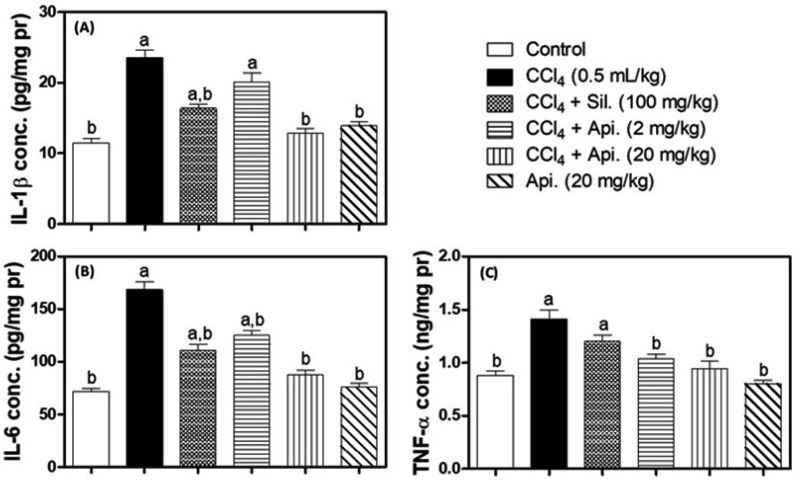
Effect of Apigenin on Inflammatory Markers: IL-1β (Panel (**A**)), IL-6 (Panel (**B**)), and TNF-α (Panel (**C**)) in CCl_4_-induced hepatic fibrosis in mice. The data are presented as mean standard deviation (n = 8). Statistical analysis was carried out using one-way ANOVA followed by Tukey’s as a post-hoc test. **CCl_4_**; carbon tetrachloride, **Sil.**; Silymarin, **Api.**; Apigenin, **pr**; protein, **conc.**; concentration. **a**: significantly different from the corresponding control at *p* < 0.05; **b**: significantly different from the corresponding CCl_4_-challenged group at *p* < 0.05.

Pro-inflammatory biomarkers such as IL-1β, IL-6, and TNF-α in liver homogenates were determined by using ELISA (Figure 4). The exposure to CCl_4_ caused a nearly two-fold rise in IL-1β and IL-6 concentrations (Figure 4A,B). In contrast, the silymarin treatment improved the IL-1β and IL-6 concentrations by about 30% as compared to the CCl_4_-intoxicated rats, whereas the treatment with a low dose of Apigenin (2 mg/kg) significantly reduced the IL-6 level (25%) in comparison with the CCl_4_-challenged group, while failing to significantly reduce the IL-1β concentration. However, the high dose of apigenin (20 mg/kg) had no significant effect on either interleukin concentration compared to the corresponding control group. CCl_4_ intoxication significantly increased TNF-α levels by 60% in comparison with the normal control group. Furthermore, silymarin therapy failed to attenuate this CCl_4_ effect because the concentrations of TNF-α in the treated animals were (36%) higher than those in the normal control group, a substantial increase. Remarkably, animals given apigenin (2 and 20 mg/kg) showed a marked reduction in TNF-α concentrations in hepatic homogenates to the point where they did not differ significantly from the normal control group. Likewise, apigenin alone did not cause a statistically significant alteration in any evaluated inflammatory cytokine concentrations in comparison to the normal control group (Figure 4C).

### 3.5. Immunohistochemical Assessment of Angiogenic Markers

#### 3.5.1. Tissue Expression of VEGF

**Figure 5:**
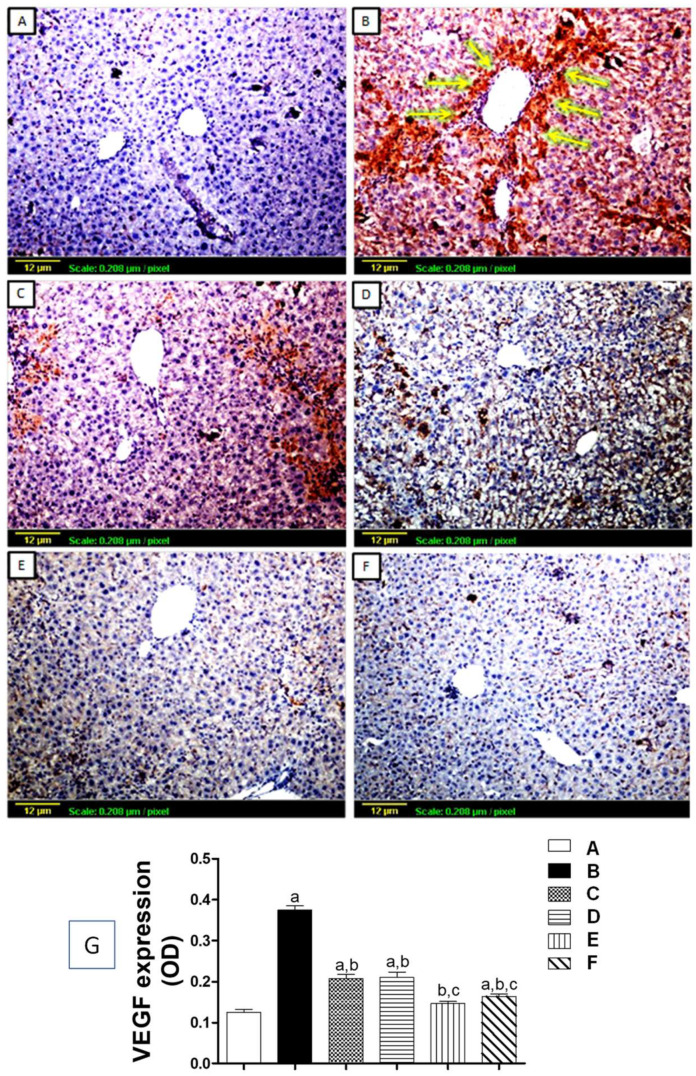
Expression of vascular endothelial growth factors (VEGF) antigen by immunohistochemical staining (×100). VEGF protein can be visualized by its brown color. (**A**) Control group showing minimal expression (+) through the hepatic tissue; (**B**) CCl_4_-exposed group showing extensive (++++) interlobular VEGF protein deposition and pericentral vein, appearing as intense brown staining **(yellow arrows)**. (**C**) Silymarin-treated group showing moderate (+++) VEGF expression, (**D**) Low-dose apigenin-treated group showing a moderate degree of VEGF protein deposition (+++); (**E**) High-dose apigenin-treated group showing minimal VEGF expression (++); (**F**) Apigenin-alone-treated group showing minimal expression in the hepatic tissue similar to the control (+). (**G**) Quantitative analysis of VEGF expression as Optical Density (OD). Statistical analysis was carried out using one-way ANOVA followed by Tukey’s as a post-hoc test. a: significantly different from the control at *p* < 0.05; b: significantly different from the CCl_4_-challenged group at *p* < 0.05; c: significantly different from the CCl_4_ + silymarin-treated group at *p* < 0.05.

The expression of VEGF protein was estimated using immunohistochemical staining (Figure 5). As demonstrated in Figure 5A, the control group showed minimal expression for VEGF antigen throughout the hepatic tissue. On the other hand, the CCl_4_-challenged group exhibited extensive VEGF protein expression, appearing as intense, brown-stained content around the central vein and in between hepatic lobules (Figure 5B), with a significant increase of OD as compared to the control (Figure 5G). Animals treated with silymarin or low-dose apigenin (2 mg/kg) presented with moderate VEGF expression in hepatic sections (Figure 5C,D). Both showed a significant reduction in OD in comparison to the CCl_4_-challenged group (Figure 4G), while with high dose apigenin (20 mg/kg), the expression of VEGF was significantly diminished (Figure 5E,G) compared to both CCl_4_-challenged and silymarin-treated groups. Moreover, apigenin treatment alone had no effect on the hepatic tissue’s expression of VEGF, and the staining pattern almost resembled that of the normal control group (Figure 5F).

#### 3.5.2. Tissue Expression of CD34

**Figure 6:**
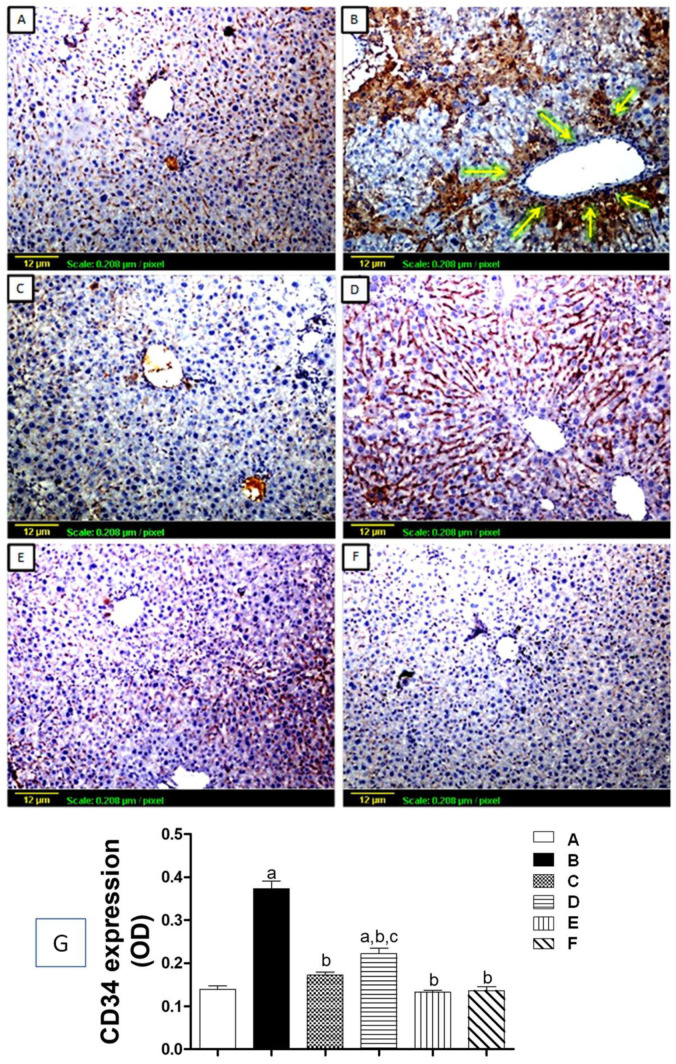
Expression of hematopoietic progenitor cell (CD34) antigen by immunohistochemical staining (×100). CD34 can be visualized by the brown color: (**A**) Control group showing minimal expression (+) through the hepatic tissue; (**B**) CCl_4_-exposed group showing intense expression (++++) of CD34 pericentral and interlobular (yellow arrows); (**C**) Silymarin-treated group showing mild CD34 expression (++); (**D**) Low-dose apigenin-treated group showing a moderate degree of expression (+++); (**E**) CCl4 + High-dose apigenin-treated group showing mild expression (++); (**F**) Apigenin-alone-treated group showing minimal expression (+) of CD34. (**G**) Quantitative analysis of CD34 expression as Optical Density (OD). Statistical analysis was carried out using one-way ANOVA followed by Tukey’s as a post-*hoc* test. **a**: significantly different from the control at *p* < 0.05; **b**: significantly different from the CCl_4_-challenged group at *p* < 0.05; c: significantly different from the CCl_4_ + silymarin-treated group at *p* < 0.05.

Vascular endothelial cell antigen (CD34) expression was also assessed by immunohistochemical staining of liver sections (Figure 6). In Figure 6A, the immune response to the sections of liver tissue of the normal control group shows a low level of CD34 antigen expression. On the other hand, tissue sections from the CCl_4_-challenged group showed a lot of brown staining around the central vein and between the lobules, which showed that CD34 was more significantly expressed compared to the control group (Figure 6B,G). The silymarin and high-dose apigenin (20 mg/kg) treatment groups both showed low levels of CD34 expression that were not significantly different from the control group (Figure 6C,E,G), although the apigenin group (2 mg/kg) demonstrated only moderate CD34 expression (Figure 6D), and the group that received only apigenin treatment resembled normal hepatic tissues (Figure 6F).

## 4. Discussion

Hepatic fibrosis is a complex fibrogenic and inflammatory process that develops due to the excessive accumulation of extracellular matrix proteins, including collagen. Hepatic fibrosis occurs in the majority of chronic liver disorders. It is well recognized now that hepatic fibrosis and steatosis might be reversible, if the hepatic architecture has not undergone significant alterations [2,8,9]. Due to liver fibrosis, there may be an increased risk of cirrhosis and possibly hepatocellular carcinoma. Untreated liver fibrosis will progress to irreversible cirrhosis that is characterized by a high risk of mortality, and transplantation is the only viable treatment [7,44]. The objectives of the present investigation were to ascertain whether apigenin might have anti-fibrotic properties against CCl_4_-induced hepatic fibrosis in mice and to examine potential molecular mechanisms underlying the inhibition of angiogenesis. Carbon tetrachloride (CCl_4_) is a frequently used hepatotoxic chemical for the induction of liver fibrosis in animal models [45,46]. In the liver, CCl_4_ causes inflammation, oxidative damage, fatty tissue degradation, and fibrosis. The liver serves as the primary location for CCl_4_ metabolism. The primary site of CCl_4_ metabolism is the liver, where cytochrome P450 enzymes convert it to a toxic metabolite. Metabolism of CCl_4_ by liver microsomal enzymes yields tri-chloromethyl radicals (CCl_3_**^−^**). These generated CCl3 radicals then react with vital intracellular molecules, which ultimately leads to lipid peroxidation and oxidative damage, finally impairing key physiological processes and resulting in altered cell functions. Consequently, generalized hepatic damage occurs, and fibrosis develops as a part of the healing process for this chronic injury [47]. In the current investigation, we have used silymarin as a standard reference drug. Several preclinical and clinical studies have shown that silymarin possesses well-established antioxidant, antifibrotic, anti-inflammatory, and hepatoprotective effects. The protective effects of silymarin on liver cells are multifaceted. Silymarin functions as a free radical scavenger and modifies the activity of enzymes that cause cellular degeneration, fibrosis, and cirrhosis. In addition, silymarin’s antifibrogenic effect has been demonstrated in non-human primates exposed to alcohol for a prolonged period of time in an animal model of alcohol-induced hepatic fibrosis [48]. In the current investigation, CCl_4_ clearly induced liver injury, which was confirmed by the hepatotoxicity biomarkers and histological analysis. However, treatment with silymarin or apigenin (2 and 20 mg/kg) considerably decreased the serum concentrations of AST and ALT. Furthermore, the apigenin (20 mg/kg) significantly improved the serum concentrations of TC, TG, and TB, which ultimately markedly improved the metabolic function. Moreover, as evidenced by the microscopic imaging, the investigated treatments were able to preserve the hepatocellular architecture and lower collagen deposition to levels similar to those in the normal control group. The efficacy of apigenin is comparable with the standard drug silymarin. Our outcomes were consistent with the previous studies that demonstrated the hepato-protective potential of apigenin against chemically induced hepatotoxicity by different chemicals. According to Ali et al. (2014), apigenin protected the liver from N-nitrosodiethylamine (NDEA)-induced hepatotoxicity [49]. Serum levels of liver enzymes were found to have dropped significantly, and the microscopic structure of the liver was found to have improved. A similar conclusion was reported by Zhou et al. (2017), who discovered that apigenin could mitigate liver toxicity experimentally induced by the administration of D-galactosamine and lipopolysaccharide [50]. In a fibrotic liver, the accumulation of reactive free radicals can damage vital biological molecules. As an illustration, the production of lipid peroxides by the oxidation of unsaturated fatty acids results in an increase in the concentration of MDA. In addition, reactive radicals would increase inflammatory mediators and initiate an inflammatory response. The eventual effect would be damaged mitochondria and nuclei, impaired cellular functions, and finally cell necrosis [13]. A healthy liver has a strong capacity to eliminate different oxidants. For instance, the CAT enzyme breaks down free radical hydrogen peroxide molecules into water and oxygen. Moreover, electron receptor molecules like GSH interact with free radicals to neutralize their harmful effects [51,52]. In the current investigation, chronic CCl_4_ exposure diminished GSH content, decreased CAT enzyme activity, and increased MDA concentrations, which is strong evidence of oxidative stress. Silymarin treatment significantly reduced the MDA concentration and increased the GSH content and CAT activity. Nevertheless, apigenin treatment (2 and 20 mg/kg) markedly decreased CCl_4_-induced oxidative stress. This was manifested by a dose-related enhancement of GSH content and CAT activity, accompanied by a reduction in MDA concentration. The apigenin (20 mg/kg) treatment brought all oxidative stress markers back to normal in CCl_4_-toxicated mice. Intriguingly, apigenin (20 mg/kg) treatment dramatically increased GSH and CAT concentrations in normal control mice. These results provided evidence that apigenin can prevent the oxidative stress that leads to inflammation and worsens fibrosis from progressing. Importantly, this antioxidant activity was consistent with earlier research by Goudarzi et al., 2021, who discovered that apigenin significantly inhibited lipid peroxidation and boosted the antioxidant defense mechanisms in experimental hepatocellular carcinogenesis [53]. Moreover, apigenin was believed to be effective in the prevention of human diseases caused by oxidative stress and the generation of free radicals [54]. Furthermore, the results of our study are consistent with earlier research that supported the role of various natural flavonoids as antioxidants for the prevention of CCl_4_-induced hepatic fibrosis [55]. Morin, Xiaochaihutang, and Hyperoside are a few examples of flavonoids as antioxidants that showed protective effects against CCl_4_-induced liver toxicity [56,57,58]. Repeated and persistent liver damage leads to liver fibrosis.

Inflammation has a vital role in the pathology of hepatic fibrosis. Injured hepatocytes will stimulate the innate immune system, leading to the activation of macrophages and chemokines. Nitric oxide and inflammatory cytokines are produced by monocytes and macrophages, which play a role in inflammation [59,60]. Moreover, Kupffer cells release powerful fibrosis promoters’ inflammation mediatory cytokines, for example TNF-α, IL-1β, and IL-6 (Batusic et al., 2011). As a result, hepatic stellate cells (HSCs) will act as myofibroblasts and proliferate to produce extracellular matrix (ECM) proteins. It is now understood that the ECM contains powerful damage-associated molecular patterns (DAMPs) with immune-active peptides. In addition, a variety of cytokines, chemokines, and growth factors that can all influence immune responses are anchored by the ECM. Moreover, the ECM creates a favorable pro-fibrogenic loop that increases the production of cytokines and chemokines and intensifies inflammatory activity [21,61]. The current investigation found that CCl_4_ exposure considerably augmented the levels of IL-6, TNF-α, and IL-1β in tissue homogenates, indicating an intensive inflammatory response after chronic damage. As expected, silymarin treatment improved IL-1β and IL-6 concentrations by about 30% as compared to the CCl_4_-intoxicated rats. Surprisingly, silymarin therapy failed to attenuate this CCl_4_ effect because the concentrations of TNF-α in the treated animals were (36%) higher than those in the normal control group, a substantial increase. Conversely, apigenin treatment (20 mg/kg) significantly reduces the levels of IL-6, TNF-α, and IL-1β as compared to the normal control group, which indicates the anti-inflammatory effect of apigenin against CCL_4_-induced inflammation, hepatotoxicity, and fibrosis. It is noteworthy that several previous investigations also revealed the anti-inflammatory effect of apigenin [29,31,62]. These studies revealed that apigenin inhibits the production of pro-inflammatory mediators such as IL-1β, TNF-α, and IL-6, which suggests the anti-inflammatory properties of apigenin. In addition, apigenin is regarded as an effective agent to treat and prevent osteoarthritis and other inflammatory conditions. Furthermore, the present findings are consistent with previous studies that showed anti-inflammatory agents were effective in preventing liver fibrosis. Natural flavonoids such as quercetin, pinocembrin, naringenin, and oroxylin A are some examples that have an anti-inflammatory effect against CCl_4_-induced liver toxicity [63,64,65,66,67].

As previously noted, the accumulation of the ECM will cause tissue hypoxia and activate proangiogenic factors, which lead to the formation of new vessels. These newly formed vessels, however, are too immature to resolve tissue hypoxia and would facilitate more expression of inflammatory cells, resulting in further deterioration of hepatic damage [23,68]. A transmembrane phosphoglycoprotein marker of vascular endothelial progenitor cells called CD34 has been shown to be useful in the detection of angiogenesis [69,70]. On the other hand, Park et al. (2015) reported that VEGF is a major contributor to fibrogenesis and portal hypertension because its overexpression accelerates the process of ECM deposition [33]. In the present investigation, immuno-histochemical analysis revealed massive expression of both VEGF and CD34 in CCl_4_-intoxicated samples, indicating the presence of angiogenesis. Mice treated with silymarin showed a low level of VEGF and CD34 expression in hepatic tissues as compared with the CCl_4_-intoxicated group. Contrarily, apigenin treatment (2 and 20 mg/kg) decreases these expressions in a dose-related manner. These outcomes are in line with those of Fu et al., 2022, who discovered that apigenin can inhibit VEGF expression, HIF-1α expression, and suppress angiogenesis in vivo for several cancer cells [71]. Furthermore, anti-angiogenic agents have also been demonstrated to prevent the development of experimental hepatic fibrosis [21,72].

## 5. Conclusions

**Figure 7:**
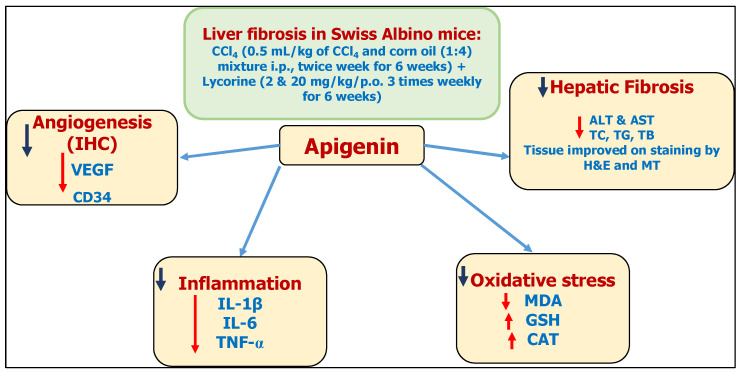
A schematic diagram of apigenin’s antifibrotic effects on CCl_4_-induced hepatic fibrosis in mice.

Finally, the current investigation has demonstrated that apigenin has hepatoprotective and antifibrotic properties. Mechanistically, apigenin attenuated oxidative stress by restoring GSH content and CAT activity as well as normalizing lipid peroxidation. In addition, it mitigated liver inflammation by reducing the expression of proinflammatory cytokines IL-6, IL-1β, and TNF-α. Moreover, it inhibited the proangiogenic factor VEGF and CD34, preventing pathological angiogenesis. These proposed mechanisms have been summarized as shown in Figure 7. However, studying the molecular pathways underlying apigenin’s antifibrotic effects is necessary because hepatic fibrosis is an extremely complex condition. Besides, more studies are required to confirm the clinical use of apigenin treatment in hepatic fibrogenesis patients. The obtained experimental effects of apigenin in improving liver fibrosis need further studies in clinical settings to judge its safety and efficacy.

## Figures and Tables

**Table 1:** Effect of apigenin on serum alanine aminotransferase (ALT) and serum aspartate aminotransferase (AST) activities, level of serum total cholesterol (TC), serum triglycerides (TG), and serum total bilirubin (TB) levels in CCl_4_-induced hepatic fibrosis in mice.

Groups	Treatments	ALT (U/L)	AST (U/L)	TC (mg/dL)	TG (mg/dL)	TB (mg/dL)
Group-1	Normal Control	19.83 ± 2.76	33.92 ± 5.67	82.17 ± 7.64	97.27 ± 5.93	0.31 ± 0.04
Group-2	CCl_4_ Control	120.67 ^a^ ± 8.94	148.08 ^a^ ± 9.85	159.2 ^a^ ± 10.02	204.1 ^a^ ± 12.48	1.72 ^a^ ± 0.14
Group-3	CCl_4_ + Silymarin (100 mg/kg)	35.16 ^a,b^ ± 5.49	56.32 ^a,b^ ± 5.76	92.55 ^b^ ± 7.50	120.72 ^a,b^ ± 6.5	0.59 ^a,b^ ± 0.08
Group-4	CCl_4_ + Apigenin (2 mg/Kg)	86.03 ^a,b,c^ ± 9.62	97.98 ^a,b,c^ ± 6.25	158.15 ^a,c^ ± 7.62	202.4 ^a,c^ ± 10.2	1.06 ^a,b,c^ ± 0.09
Group-5	CCl_4_ + Apigenin (20 mg/Kg)	46.15 ^a,b^ ± 4.97	73.55 ^a,b,c^ ± 5.28	111.6 ^a,b,c^ ± 8.65	157.8 ^a,b,c^ ± 7.68	0.76 ^a,b,c^ ± 0.10
Group-6	Apigenin Alone (20 mg/Kg)	21.30 ^b,c^ ± 3.92	32.50 ^b,c^ ± 6.63	80.25 ^b^ ± 4.04	98.42 ^b,c^ ± 8.35	0.31 ^b,c^ ± 0.04

Data are displayed as Mean ± S.D. (*n =* 8); a: significantly different from the corresponding control at *p* < 0.05; b: significantly different from the corresponding CCl_4_-challenged group at *p* < 0.05; c: Significantly different from the corresponding silymarin-treated group at *p* < 0.05; CCl_4_; carbon tetrachloride.

## Data Availability

Not applicable.
